# Direct Identification of Bacteria in Positive Blood Culture Bottles by Matrix-Assisted Laser Desorption Ionisation Time-of-Flight Mass Spectrometry

**DOI:** 10.1371/journal.pone.0008041

**Published:** 2009-11-25

**Authors:** Bernard La Scola, Didier Raoult

**Affiliations:** Pôle des Maladies Infectieuses, Assistance Publique-Hôpitaux de Marseille and URMITE UMR CNRS-IRD 6236, IFR48, Faculté de Médecine, Université de la Méditerranée, Marseille, France; Charité-Universitätsmedizin Berlin, Germany

## Abstract

**Background:**

With long delays observed between sampling and availability of results, the usefulness of blood cultures in the context of emergency infectious diseases has recently been questioned. Among methods that allow quicker bacterial identification from growing colonies, matrix-assisted laser desorption ionisation time-of-flight (MALDI-TOF) mass spectrometry was demonstrated to accurately identify bacteria routinely isolated in a clinical biology laboratory. In order to speed up the identification process, in the present work we attempted bacterial identification directly from blood culture bottles detected positive by the automate.

**Methodology/Principal Findings:**

We prospectively analysed routine MALDI-TOF identification of bacteria detected in blood culture by two different protocols involving successive centrifugations and then lysis by trifluoroacetic acid or formic acid. Of the 562 blood culture broths detected as positive by the automate and containing one bacterial species, 370 (66%) were correctly identified. Changing the protocol from trifluoroacetic acid to formic acid improved identification of *Staphylococci*, and overall correct identification increased from 59% to 76%. Lack of identification was observed mostly with viridans streptococci, and only one false positive was observed. In the 22 positive blood culture broths that contained two or more different species, only one of the species was identified in 18 samples, no species were identified in two samples and false species identifications were obtained in two cases. The positive predictive value of bacterial identification using this procedure was 99.2%.

**Conclusions/Significance:**

MALDI-TOF MS is an efficient method for direct routine identification of bacterial isolates in blood culture, with the exception of polymicrobial samples and viridans streptococci. It may replace routine identification performed on colonies, provided improvement for the specificity of blood culture broths growing viridans streptococci is obtained in the near future.

## Introduction

Currently, blood culture is likely the most significant specimen type used for the diagnosis of bacterial infections, especially for bloodstream infections. Automates perform continuous monitoring of bacterial growth, which ensures quick reports to the physicians. When a blood culture bottle is identified as growing bacteria the automate, presumptive identification is based on Gram staining, which allows classification of bacteria as either cocci or bacilli and as Gram-positive or Gram-negative. This information is given to clinicians in order to adapt presumptive antibiotic therapy. Detection of particular organisms is sometimes indicative of an aetiology, i.e., all blood cultures positive for Gram-positive cocci in chains will orient diagnosis toward streptococcal endocarditis. Blood culture medium would then be subcultured on agar plates in order to obtain bacteria colonies that would be subjected to identification. However, conventional identification is based on time-consuming procedures that hamper proper management of patients with respect to antibiotic and supportive treatments. Routine bacterial identification is based on phenotypic tests, including Gram staining, culture and growth characteristics and biochemical patterns. Complete identification is routinely achieved within two days, but may be longer for fastidious or atypical organisms. As this delay between blood culture sampling and definitive identification of the organism responsible for the bacteremia and antibiotic susceptibility testing is long, the usefulness of sampling blood cultures at admission in emergency departments has been recently questioned [1]–[3]. Many improvements have been proposed to speed up the process of detection and identification. In fact, the most rapid detection systems based on direct detection of the most commonly encountered bacteria in blood by real-time PCR are currently being evaluated, but they do allow for evaluation of antibiotic susceptibility [4], [5]. The other systems are based on quick identification of bacteria that have grown in blood culture bottles. First of all, improvements in culture media and detection of growth procedures have reduced these delays. The most recent generation of automates can detect even small bacterial growth [6]–[8]. When growth is detected by the automate, it is possible to perform direct identification of bacteria by molecular biology, such as universal amplification and sequencing [9], nucleic acid-based fluorescence hybridisation probes, such as FISH [10]–[12], DNA microarrays [13] or molecular detection amplification and specific probes [14]. These last systems are usually not open and only allow detection of one or a few specific targets; however, they may provide no information about presumptive antibiotic susceptibility (i.e., detection of MRSA) [15], [16]. These procedures are efficient but are expensive and/or require high qualifications of bacteriology technicians. The same procedures may be used on isolated colonies or even less complex systems for defined microorganisms, such as the use of monoclonal or polyclonal specific antibodies, which are currently used for agents commonly encountered in bacteremia, such as *Staphylococcus aureus* or *Streptococcus pneumoniae* [17], [18]. Among the most recent procedures for rapid identification, bacterial identification based on peptide spectra obtained by matrix-assisted laser desorption ionisation time-of-flight (MALDI-TOF) mass spectrometry has been proposed and recently was successfully used for routine identification of bacterial colonies in a university 4000-bed hospital [19]. Identification is quick; bacterial identification is obtained on the day the bacterial colonies grow on subculture and thus the result is obtained approximately one day earlier than with the conventional procedure. As positive blood cultures represent a suspension of bacteria and blood cells, we thought to separate bacteria and then subject them to MALDI-TOF mass spectrometry. In the present work, we evaluated the performance of MALDI-TOF mass spectrometry for direct identification of bacteria in blood culture bottles detected as positive by the automate.

## Materials and Methods

### Blood Culture System and Identification of Isolates

Our laboratory is equipped with Bactec 9240 automates, and blood samples were taken in Bactec Plus aerobic and Lytic 10 anaerobic blood culture bottles and processed in the automate according to the manufacturer's recommendations. When a blood culture bottle was detected as positive, the blood culture broth was deposited on a glass slide to be subjected to Gram staining and was subcultured onto Chocolat polyvitex and Columbia sheep blood agar (Biomerieux, Marcy l'Etoile France) under aerobic and anaerobic atmospheres. Study began by using protocol 1 which was modified as protocol 2 in the second part of the study. All positive blood culture bottles (one bottle per patient in the case of several bottles are positive for the same patient during protocol 1) were prospectively included over eight months. During the period of protocol 1, the isolates were recovered after subculture, semi-automated Gram staining (Aerospray Wiescor apparatus, Elitech, Signes, France) and determination of catalase and oxidase activities. Isolates were inoculated into the appropriate VITEK identification strip using the VITEK 2 apparatus (BioMérieux) or API ANA identification strip for anaerobes (BioMérieux). Comparison using MALDI-TOF mass spectrometry on colonies was performed as described by Seng et al. [19]. During the period of protocol 2, isolates were identified by MALDI-TOF mass spectrometry only. As for direct identification using MALDI-TOF mass spectrometry directly on blood culture bottles samples were anonymized and results obtained by using the protocol presented herein not transmitted to clinicians in charge of patients, we did not ask for informed consent of patients. This process was submitted and approved by the Comite d'Ethique de l'IFR48.

### Preparation of Samples for Mass Spectrometry

#### Protocol 1

Positive blood culture broth (1 ml) was centrifuged at 500 rpm for 15 minutes in order to remove red blood cells. The supernatant was then centrifuged at 14000 rpm for 20 minutes. The supernatant was discarded, and the pellet was resuspended in 1 ml of sterile distilled water, vortexed, and then centrifuged again at 14000 rpm for 20 minutes. After centrifugation, 5 µl of acetonitrile (AN) and 5 µl of 20% trifluoroacetic (TFA) acid were mixed, added to the pellet and incubated for 15 minutes at room temperature. After a short centrifugation at 14000 rpm for 2 minutes, 2 µl of supernatant was spotted onto a MALDI-TOF MTP 384 target plate (Bruker Daltonik GmbH, Leipzig, Germany). Four such deposits were made for each isolate. The preparation was overlaid with 2 µL of matrix solution [saturated solution of α-HCCA (alpha-cyano-4-hydroxycinnamic acid) in 50% AN and 2.5% TFA]. The matrix-sample was crystallised by air drying at room temperature for 5 minutes.

#### Protocol 2

Positive blood culture broth (1 ml) was centrifuged at 500 rpm for 5 minutes in order to remove red blood cells. The supernatant was then centrifuged at 14000 rpm for 5 minutes. The supernatant was discarded, and the pellet was resuspended in 1 ml of sterile distilled, water, vortexed, and then centrifuged again at 14000 rpm for 5 minutes. After centrifugation, 5 µl of formic acid was added to the pellet and incubated for 5 minutes at room temperature. Then, 5 µl of acetonitrile was added, and after vortexing and centrifugation at 14000 rpm for 2 minutes, 2 µl of supernatant was deposited on a MALDI-TOF target plate and processed as described above.

### Mass Spectrometry

Measurements were performed with an Autoflex II mass spectrometer (Bruker Daltonik) equipped with a 337-nm nitrogen laser. Spectra were recorded in the positive linear mode (delay: 170 ns; ion source: 1; (IS1) voltage: 20 kV; ion source 2 (IS2) voltage: 18.5 kV; lens voltage: 7 kV; mass range: 2 kDa to 20 kDa). Each spectrum was obtained after 675 shots in automatic mode at a variable laser power, and the acquisition time ranged from 30–60 seconds per spot. Data were automatically acquired using AutoXecute acquisition control software. The spectra of the four spots for each isolate were imported into BioTyper™ version 2.0 software (Bruker Daltonik GmbH) and analysed by standard pattern matching (with default parameter settings) against the spectra of 2,881 species used as references in the BioTyper™ database (these spectra are an integrated part of the BioTyper™ software version, as updated in June 2008). The method of identification included the mass to charge ratio (m/z) from 3 to 15 kDa. For each spectrum, no more than 100 peaks were taken into account, and these peaks were compared with peaks in the database. The 15 bacterial species exhibiting the most similar peptide pattern with the isolate were ranked by their identification score.

### Criteria for Identification of Isolates

For MALDI-TOF analysis, we adapted the score values proposed by Sengh et al.: an isolate was considered correctly identified by MALDI-TOF when ≥2/4 spectra had a score ≥1.9 or 4/4 had a score ≥1.2.

## Results

### Positive Blood Culture Bottles

During the study period, 599 blood culture bottles corresponding to 559 patients were tested. Among these, 15 led to the isolation of strains for which no identification could be obtained by phenotypic or MALDI-TOF mass spectrometry on colonies; they were thus not considered for analysis. Among the 584 remaining positive blood cultures, 22 grew more than one species and the other 562 grew a single species.

### Concordant MALDI-TOF Identification (Table 1)

During the period of protocol 1, 322 positive blood culture bottles were analysed over a five-month period. Among these, 59% yielded identical identification to routine phenotypic tests at the species level. There was a clear difference between Gram-positive and Gram-negative bacteria, as 94% of Gram-negative bacteria were identified, whereas only 37% of Gram-positive bacteria were identified. Among these, *Streptococcus* sp. were poorly identified (4/25, 16%). Protocol 2, which was tested during three additional months, clearly improved the results of identification. For Gram-negative bacteria, the results were comparable or slightly worse to protocol 1, but for Gram-positives, this protocol significantly improved identification, with 67% identification. This improvement was mostly effective for *Staphylococcus* sp., as *Streptococcus* sp. remained poorly identified (4/17, 23%). For most of the represented Gram-positive species, protocol 2 also improved the mean best score obtained, as it increased from 1.43 and 1.49 to 2 and 2 for *S. aureus* and *Staphylococcus epidermidis*, respectively (Table 1).

**Table 1:** The results according to the species and the protocol used for preparation of broth before deposit for MS testing of MS identification on 599 monomicrobial positive blood culture bottles tested.

Species	Protocol 1			Protocol 2			
	Tested (n = )	Identified (%)	Mean best score	Tested (n = )	Identified (%)	Mean best score	p*
**Gram-negative**							
*Escherichia coli*	71	68 (96%)	2.02	34	31 (94%)	2.1	
*Klebsiella pneumoniae*	10	9 (90%)	2.05	18	17 (94%)	1.9	
*Klebsiella oxytoca*	4	4 (100%)		3	3 (100%)		
*Raoultella planticola*	1	1 (100%)					
*Raoultella ornithinolytica*	0			1	1 (100%)		
*Enterobacter aerogenes*	2	2 (100%)		10	8 (80%)		
*Enterobacter cloacae*	4	4 (100%)		9	7 (77%)		
*Serratia marcescens*	3	3 (100%)		5	4 (80%)		
*Morganella morganii*	1	1 (100%)		1	1 (100%)		
*Citrobacter koseri*	2	2 (100%)		2	2 (100%)		
*Proteus mirabilis*	0			4	4 (100%)		
*Pantoea agglomerans*	1	0 (0%)					
*Salmonella enterica*	3	2 (66%)		0			
*Pseudomonas aeruginosa*	12	12 (100%)	2.26	3	3 (100%)		
*Acinetobacter baumannii*	2	1 (50%)		0			
*Pseudomonas fluorescens*	0	0 (0%)		0			
*Stenotrophomonas maltophilia*	0			1	1 (100%)		
*Haemophilus influenzae*	2	0 (0%)		1	1 (100%)		
*Haemophilus parainfluenzae*	2	2 (100%)		0			
*Neisseria meningitidis*	2	1 (50%)		0			
*Aeromonas hydrophila*	0			1	1 (100%)		
*Achromobacter ruhlandi*	0			1	1 (100%)		
*Ochrobactrum anthropi*	0			4	0 (0%)		
*Bacteroides fragilis*	3	3 (100%)		0			
*Bacteroides ovatus*				1	1 (100%)		
*Bacteroides vulgatus*				1	1 (100%)		
**Gram-negative**	**125**	**117 (94%)**		**100**	**87 (87%)**		**0.09 NS**
**Gram-positive**							
*Staphylococcus aureus*	50	20 (40%)	1.43	21	15 (58%)	2	
*Staphylococcus epidermidis*	59	19 (32%)	1.49	71	57 (80%)	2	
*Staphylococcus hominis*	16	9 (56%)		8	6 (75%)		
*Staphylococcus warneri*	9	2 (22%)		1	1 (100%)		
*Staphylococcus haemolyticus*	6	3 (50%)		4	1 (25%)		
*Staphylococcus xylosus*	1	0 (0%)		0			
*Staphylococcus lugdunensis*	2	1 (50%)		0			
*Staphylococcus capitis*	5	3 (60%)		3	2 (33%)		
*Staphylococcus caprae*	1	0 (0%)		0			
*Staphylococcus cohnii*	2	1 (50)		0			
*Staphylococcus saprophyticus*	1	0 (0%)		1	0 (0%)		
*Micrococcus* sp.	4	0 (0%)		2	1 (50%)		
*Enterococcus faecalis*	10	9 (90%)		5	3 (60%)		
*Enterococcus faecium*	3	1 (33%)		4	3 (75%)		
*Streptococcus pneumoniae*	9	3 (33%)		3	2 (66%)		
*Streptococcus pyogenes*	2	1 (50%)		1	1 (100%)		
*Streptococcus sanguis*	2	0 (0%)		0			
*Streptococcus intermedius*	1	0 (0%)		0			
*Streptococcus agalactiae*	3	0 (0%)		1	1 (100%)		
*Streptococcus mitis*	3	0 (0%)		2	0 (0%)		
*Streptococcus salivarius*	2	0 (0%)		0			
*Streptococcus anginosus*	3	0 (0%)		0			
*Streptococcus constellatus*	0			3	0 (0%)		
*Streptococcus oralis*	0			2	0 (0%)		
*Streptococcus haemolyticus*	0			1	0 (0%)		
*Streptococcus gallolyticus*	0			4	0 (0%)		
*Gemella morbillorum*	1	0 (0%)		0			
*Corynebacterium jeikeium*	1	0 (0%)					
*Corynebacterium ulcerans*	1	0 (0%)					
*Corynebacterium* sp.	0			2	0 (0%)		
*Bacillus cereus*	0			1	1 (100%)		
**Gram-positive**	**197**	**72 (37%)**		**140**	**94 (67%)**		**<10^−2^**
**All**	**322**	**189 (59%)**		**240**	**181 (76%)**		**<10^−2^**

*Chi-square test.

### Erroneous MALDI-TOF Identification

Only one isolate in one blood culture sample growing a unique species prepared with protocol 1 and growing *Serratia marcescens* was misidentified as *Aeromonas hydrophila*, with 2/4 positive spots and a score >1.9. The major cause of erroneous identification was the presence of two or more species in the same bottle. A mixture of species occurred in 22 blood culture bottles (Table 2). For two of these samples, no identification was obtained. For 18 samples, only one of the two isolates was identified. In the two remaining cases, including one with three different species in the mixture, MS identification was erroneous.

**Table 2:** The results of MS identification of the 22 blood culture bottles containing two different species (* mixture of species resulting in false positive results).

Species 1	Species 2	MS identification
*E. coli*	*S. epidermidis*	*E. coli*
	*E. faecalis*	*E. coli*
	*E. faecalis*	*E. coli*
	*K. pneumoniae*	*E. coli*
	*P. mirabilis*	*E. coli*
	*E. faecalis*	*E. faecalis*
	*A. baumannii*	*A. baumannii*
	*S. pneumoniae*	*S. pneumoniae*
*S. epidermidis*	*S. marcescens*	S. aureus*
	*S. haemolyticus*	*S. haemolyticus*
	*S. haemolyticus*	*S. epidermidis*
	*E. faecalis*	*E. faecalis*
	*S. aureus*	*S. epidermidis*
*P. aeruginosa*	*E. faecalis*	*E. faecalis*
	*S. aureus*	*S. aureus*
	*B. fragilis*	*B. fragilis*
*E. faecalis*	*S. hominis*	*E. faecalis*
	*K. pneumoniae + A. sobria*	A. culicicola*
*E. cloacae*	*E. faecium*	*E. cloacae*
	*S. aureus*	No identification
*S. aureus*	*P. avidum*	No identification
*M. morganii*	*S. pneumoniae*	*M. morganii*

## Discussion

Many studies have demonstrated that rapid antimicrobial therapy adapted to the susceptibility of bacteria responsible for bloodstream infections can reduce morbidity and mortality [20]–[23]. In this study, we tested 599 blood culture bottles identified as culture positive by our Bactec 9240 automate. The efficiency of mass spectrometry to identify crude bacteria was first established in non-medical microbiology laboratories [24] and was recently confirmed as the most promising technique for routine bacterial identification in clinical microbiology laboratories [19], [25], [26]. In the present study, two different protocols for blood culture broth preparation before MS were tested. The first protocol using TFA had been reported to be efficient on bacterial colonies [19],; however, it lacked efficiency for Gram-positive bacteria in blood culture broth (37% positive identification), whereas it allowed the identification of 94% of Gram-negative bacteria. By using formic acid instead of TFA (protocol 2), the identification of Gram-positive bacteria increased to 67% positive identification, and it remained high for Gram-negatives, with 88% positive identification. Moreover, for the identified Gram–positive bacteria, better scores were obtained with this protocol, as the mean best score for *S. aureus* and *S. epidermidis* was 2 (data not shown). In fact, a weakness of MS identification of bacteria from positive blood cultures was observed with viridans streptococci, which were not identified at all. This slight loss in efficiency of protocol 2 was mostly due to negative results observed in four blood culture bottles containing *Ochrobactrum anthropi* (Table 1).

In direct testing, we experienced only three false positive results, for a positive predictive value of 99.2%. In two of the three cases, the false positive result was due to mixtures of two and three different species in the same bottle. For the other mixtures of bacteria, identification was correct, but only one of two species was identified, even by testing four spots (Table 2). Moreover, it was impossible to predict what the species identified in the mixture would be. For example, for the three mixtures containing both *Enterococcus faecalis* and *Escherichia coli*, *E. coli* was identified in two cases but *E. faecalis* was identified in only one. This result means that Gram staining of blood culture broths after detection by the automate cannot be omitted, as Sengh et al. proposed in the case of colonies [19].

**Figure 1:**
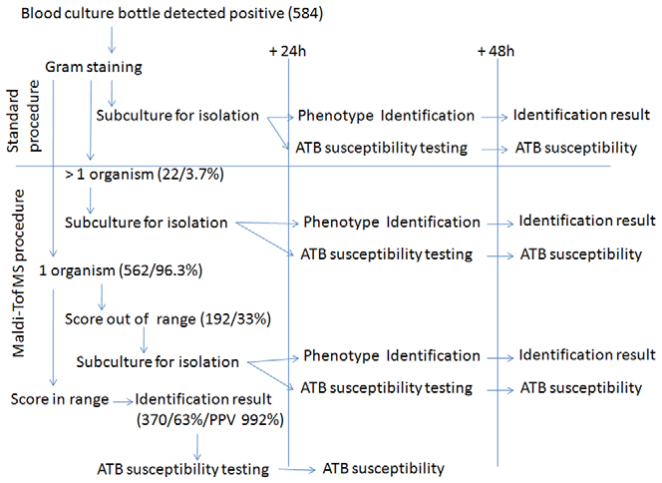
MALDI-TOF identification. Proposed MALDI-TOF MS procedure for identification and antibiotic susceptibility testing of bacteria isolated from blood culture and comparison of conventional procedure. The data obtained in the present study are indicated in parentheses.

Results of the identification by MALDI-TOF MS may be obtained approximately two hours after the automate has detected it to be positive. Without automation of the procedure, it would not be possible to perform sample preparation for MS each time a blood culture is detected positive by the automate; however, the procedure may be repeated several times a day, i.e., three times a day in our laboratory currently. As shown in Figure 1, our procedure provides identification results 48 h sooner than the conventional procedure. Moreover, by obtaining quick identification, it is possible to perform direct antimicrobial susceptibility testing by using blood culture bottle broth. This technique has been demonstrated to be efficient for antibiotic susceptibility testing with both manual and automated systems, provided that the inoculums are well calibrated [27]–[30]. With such procedures, blood culture again becomes the sample of choice upon admission of febrile patients to the emergency department. This procedure requires more identification time than direct detection in blood using a real-time PCR system as it require bacterial growth in blood culture bottle before identification [4], [5], but it allows antibiotic susceptibility testing. For quick detection of resistance to key antibiotics, it is also possible to combine MS-based identification with molecular-based antibiotic resistance prediction, such as the Cepheid Xpert MRSA/SA blood culture assay, which allows detection of MRSA and *S. aureus* in blood culture bottles detected positive by the automate [16]. This test was demonstrated to have a sensitivity of 98.3% for detection of MRSA in blood culture bottles. From our point of view, the major drawback of Cepheid technique is the cost for the unitary test, as it has to be performed on all blood culture bottles in which clusters of Gram-positive cocci are detected, whereas most of these Gram-positive cocci are coagulase-negative staphylococci, as shown in our study (191/262, 73%). The association of MS identification on blood culture bottles followed by Cepheid Xpert MRSA/SA blood culture assay on *S. aureus* growing in blood culture bottles is currently being evaluated in our laboratory with promising results. On the other hand, in a previous study on *S. aureus* using MALDI-TOF, it was observed that MRSA spectral profiles were different from methicillin-susceptible *S. aureus* [31]. Detection of beta-lactamase was also possible with ampicillin-resistant *E. coli* [32]. However, as signature peaks may shift according to experimental conditions [32], it will probably be necessary to construct a specific database of susceptible and resistant organisms growing in blood culture bottles in order to use MS profiles for antibiotic susceptibility prediction.

In the present study, the prospectively gathered data demonstrated that MALDI-TOF identification of bacteria present in blood culture bottles previously identified as positive by an automate is an efficient method for the rapid and routine identification of bacterial isolates in the clinical microbiology laboratory. This technique will probably replace conventional identification of bacteria in blood cultures, but it currently presents two major pitfalls. First, in the case of a mixture of species, only one is identified, and false identification can occur. This means that Gram staining of blood culture broth must be done in all cases. The second is that it does not allow identification of viridans streptococci. As such identification is possible from colonies growing on agar, it is likely that improvement in the preparation of bacteria before deposit could rectify this shortcoming.
